# Is Vitamin D Deficiency Related to Accumulation of Advanced Glycation End Products, Markers of Inflammation, and Oxidative Stress in Diabetic Subjects?

**DOI:** 10.1155/2015/958097

**Published:** 2015-04-27

**Authors:** K. Šebeková, M. Stürmer, G. Fazeli, U. Bahner, F. Stäb, A. Heidland

**Affiliations:** ^1^Comenius University Medical Faculty, 811 07 Bratislava, Slovakia; ^2^University of Würzburg, 97080 Würzburg, Germany; ^3^KfH Nierenzentrum Würzburg, 97080 Würzburg, Germany; ^4^Beiersdorf AG, Hamburg, Germany

## Abstract

*Objectives*. In diabetes accumulated advanced glycation end products (AGEs) are involved in the striking cardiovascular morbidity/mortality. We asked whether a hypovitaminosis D associates with an increased formation and toxicity of AGEs in diabetes. *Methods*. In 276 diabetics (160 M/116 F, age: 65.0 ± 13.4; 43 type 1,T1DM, and 233 type 2 patients, T2DM) and 121 nondiabetic controls (60 M/61 F; age: 58.6 ± 15.5 years) routine biochemistry, levels of 25-hydroxyvitamin D_3_ (25-(OH)D), skin autofluorescence (SAF), plasma AGE-associated fluorescence (AGE-FL), N^*ε*^-(carboxymethyl)lysine (CML), soluble receptor for AGEs (sRAGE), soluble vascular adhesion protein-1 (sVAP-1), high sensitive C-reactive protein (hs-CRP), and renal function (eGFR) were determined. *Results*. In the diabetics SAF and AGE-Fl were higher than those of the controls and correlated with age, duration of diabetes, and degree of renal impairment. In T2DM patients but not in T1DM the age-dependent rise of SAF directly correlated with hs-CRP and sVAP-1. 25-(OH)D levels in diabetics and nondiabetics were lowered to a similar degree averaging 22.5 ng/mL. No relationship between 25-(OH)D and studied markers except for sVAP-1 was observed in the diabetics. *Conclusion*. In diabetics hypovitaminosis D does not augment accumulation of AGEs and studied markers of microinflammation and oxidative stress except for sVAP-1.

## 1. Introduction

Advanced glycation end products (AGEs) are a heterogeneous group of compounds implicated in the pathophysiology of aging, diabetes mellitus, and chronic kidney disease (CKD). They are formed by nonenzymatic glycation of proteins, lipids, and nucleic acids and under conditions of oxidative and carbonyl stress [1, 2]. Other factors involved in accumulation of AGEs are their impaired renal removal in kidney dysfunction [3], consumption of highly heat-treated foods with an elevated AGE content [4, 5] and inhalation of tobacco smoke [6]. AGEs exert their deleterious effects directly by modifications of long-lived intra- and extracellular proteins, which affect their structural and functional properties. Cross-linking of collagen promotes vascular stiffness [7] and also injures the skeletal muscle [8]. Indirect harmful effects arise from interactions of AGEs with their receptors (particularly RAGE) at the cell membrane. RAGE activation induces nuclear transcription factors (e.g., nuclear factor kappa-B, NF-*κ*B), generation of oxygen radicals, synthesis of proinflammatory cytokines/chemokines, fibrogenic growth factors (transforming growth factor-*β*-1, TGF-beta-1), vascular adhesion molecules and cell proliferation [9], and reduction of nitric oxide (NO) formation [10]. AGEs may also interrupt key steps in reverse cholesterol transport [11]. Beside cardiovascular disturbances, AGE accumulation is linked to an enhanced cancer incidence, in part due to an AGE-induced genomic damage [12, 13].

Diabetes mellitus is associated with an excessive accumulation of AGEs [14]. Subsequently a microangiopathy (nephropathy, neuropathy, and retinopathy) and an accelerated atherosclerotic vasculopathy (including coronary heart, cerebrovascular, and peripheral artery disease) develop [15]. AGEs may directly contribute to induction or aggravation of diabetes causing progressive insulin secretory defects and pancreatic beta cell deaths [16] and by enhancing insulin resistance via decreased biological activity of glycated insulin [17].

Elevated levels of circulating AGEs such as pentosidine, N^*ɛ*^-carboxymethyllysine (CML), and AGE-associated fluorescence (AGE-Fl) were related to coronary and peripheral artery disease (PAD), renal damage, and total cardiovascular mortality in the general population [18–20], in particular in patients with type 2 diabetes [21] and end-stage renal disease [22]. The toxic effects of AGEs are partly neutralized by soluble RAGE (sRAGE), which represents the truncated form of the receptor acting as a decoy [23].

In the past several years, a noninvasive measurement of skin AGE-associated autofluorescence (SAF) has been developed. SAF is closely related to AGE accumulation in the tissues and reflects the “long term cumulative metabolic and oxidative stress.” SAF is an independent predictor of cardiovascular complications, morbidity, and mortality [24–27].

Similar to the consequences of AGE accumulation, vitamin D deficiency may be involved in numerous biochemical and clinical disturbances, besides the musculoskeletal disorders induced by secondary hyperparathyroidism [28]. Observational and prospective studies showed associations of vitamin D_3_ deficiency with cardiovascular disease [29], hypertension (via stimulation of the renin-angiotensin-aldosterone system [30–32]), vascular stiffness [33], coronary artery calcification [34, 35] heart hypertrophy, stroke [36], renal damage, autoimmune diseases (type I diabetes, multiple sclerosis, and rheumatoid arthritis) [28], infections (lowered antimicrobial peptide cathelicidin [37]), and impaired cognitive function including Alzheimer disease [38]. Vitamin D deficiency likewise seems to be involved in impaired glucose tolerance or type 2 diabetes [39]. It may predispose to an impaired insulin secretion via the vitamin D receptor (VDR) in the beta cells of the pancreas [40–42] and an impaired insulin sensitivity, resulting in insulin resistance [43–45]. In type 1 diabetes severe vitamin D deficiency predicts all-cause mortality [46]. Moreover reduced muscle function may be associated with both vitamin D deficiency [47] and AGE accumulation [8].

Data about potential relationships between vitamin D_3_ deficiency and AGE accumulation are, so far, scarce. In in vitro studies it was shown that the deleterious effects of AGE-modified albumin on endothelial cells could be prevented by coincubation with calcitriol, the active form of vitamin D [48]. In diabetic rats administration of vitamin D reduced systemic oxidative stress and the deposition of AGEs (CML) in the aortic wall [49]. In the current paper we investigated the impact of vitamin D status on the AGE levels in skin and plasma and markers of microinflammation and oxidative stress as well as on muscle function in diabetic patients to elucidate the potential interactions.

## 2. Subjects and Methods

This cross-sectional noninterventional study was conducted according to the Declaration of Helsinki and a protocol approved by the Ethics Committee of the Medical Faculty of the University of Würzburg. Signed written informed consent was obtained from all participants.

A total of 276 consenting diabetic patients (age range: 16–94 years; 18% type 1 diabetes mellitus, (DM) duration: newly diagnosed to 56 years) visiting the ambulance of the KfH-Kidney Center Würzburg and the Practice of Internal Medicine (Dr. Werner Stürmer) in Würzburg were recruited. Inclusion criteria were type 1 or type 2 DM. Control subjects (*n* = 121; age range: 16–96 years) were recruited from participants of regular check-ups in the same practice during the same time period as well as the staff. Exclusion criteria for both controls and diabetics comprised any acute illness, autoimmune diseases, malignancies, dermatosis, scars and pigment disorders, pregnancy or lactation in women, current smoking (self-reported), use of glucocorticoids, vitamin D supplements (during the last 6 months), regular visits to a solarium, and use of tanning cream (during the last 14 days). Patients with hypertension and/or diabetes were treated according to the current guidelines.

Weight and height were measured and body mass index (BMI) was calculated. SAF was measured on the volar side of the forearm using the AGE-Reader (DiagnOptics BV Groningen, Netherlands) as previously described [21]. Hand-grip muscle strength was measured using the Baseline Hydraulic Hand Dynamometer (White Plains NY, USA).

Venous blood was collected in the morning hours (7.00 to 9.00 h), after overnight fasting and analyzed for serum creatinine, haemoglobin A1c (HbA1c, HPLC method, ADAM A1c HA 8180 FAST, Axonlab, Germany), high sensitive C-reactive protein (hs-CRP, nephelometrically, Siemens reagent), and 25(OH)D (electrochemiluminescence immunoassay, ECLIA, Roche, Germany) in a certified laboratory (Labor Limbach, Heidelberg, Germany). Vitamin D deficiency was defined as 25(OH)D level <20 ng/mL, vitamin D insufficiency as 25(OH)D level 20–30 ng/mL, and vitamin D sufficiency as 25(OH)D level >30 ng/mL. Abbreviated MDRD formula was used to estimate glomerular filtration rate (eGRF).

Aliquots of plasma were stored at −80°C and transferred on dry ice to a laboratory in Bratislava for determination of total proteins (Vitros 250 analyzer, USA), AGE-associated fluorescence of plasma according to Münch et al. [50], and concentrations of CML (ELISA, Microcoat, Bernried, Germany), sRAGE (ELISA, R&D Systems, Minneapolis, MN, USA), and soluble vascular receptor adhesion protein-1 (sVAP-1, ELISA, Bender MedSystem Inc., Vienna, Austria) using commercial ELISA kits according to manufacturer's instructions.

Presence of comorbidities (hypertension (HT), coronary heart disease (CHD), and peripheral artery disease (PAD)) was tracked from documentation and was not available for 12 newly diagnosed diabetics.

### 2.1. Statistical Analysis

Data not distributed normally were logarithmically transformed for statistical analyses. Descriptive statistics are presented as percentages or means ± SD. Two sets of data were compared using two-sided Student's *t*-test, for comparison of ≥3 sets of data analysis of variance (ANOVA) with post hoc Scheffe's test was employed. Proportions were compared using chi-square test. Pearson's correlation coefficients were calculated. Multivariate analysis was performed using the General Linear Model (GLM). SPSS statistical software (v. 16.0 for Windows; SPSS, Chicago, Illinois) was used with the significance set at *P* < 0.05. The orthogonal projections to latent structures discriminant analysis (OPLS-DA, Simca v.13 software, Umetrics, Umea, Sweden) was used to identify independent variables contributing to separation between subjects with 25(OH)D deficiency and those with sufficient levels.

## 3. Results

### 3.1. Cohort Characteristics

#### 3.1.1. Nondiabetics versus Diabetic Subjects

Cohort characteristics are given in Table 1. The proportion of females and males, CML, and sRAGE concentrations did not differ significantly between diabetic and control subjects. DM patients were significantly older (*P* < 0.001) and had as expected higher BMI (*P* < 0.001), HbA1c (*P* < 0.001), SAF (*P* < 0.001), hsCRP (*P* = 0.001), AGE-associated fluorescence (*P* < 0.001), and sVAP-1 (*P* = 0.049) levels, presented higher frequency of comorbidities, a lower eGFR (*P* < 0.001), and grip strength (*P* = 0.026) in comparison to controls. To elucidate the independent effects of age and presence of diabetes, multivariate analysis using the GLM was employed. Selected independent variables did not affect significantly CML levels (Table 2).

#### 3.1.2. Impact of Type of Diabetes

Type 1 diabetic patients were younger (*P* < 0.001) than their type 2 DM counterparts (Table 1). Glycemia was similar in both cohorts (*P* = 0.98) and DM was diagnosed also for a comparable time period (*P* = 0.10). Type 1 and 2 diabetics did not differ significantly in CML, sRAGE, and sVAP-1 levels and produced a similar strength in the hand-grip test. In comparison to type 2 diabetic patients, type 1 diabetics presented lower BMI, AGE-specific fluorescence of plasma, SAF (*P* < 0.001), and hsCRP (*P* = 0.004) levels and higher eGFR (*P* < 0.001). The potential impact of the type of diabetes, its duration, and subjects' age on studied markers was estimated using the GLM. It did not select type of diabetes as independent significant contributor in either setting (Table 3). Duration of diabetes significantly and independently affected eGFR, AGE-Fl, SAF, sVAP-1, and grip strength, while aging was significantly associated with decline in eGFR and rise in AGE-Fl and SAF. However, in case of CML, sVAP-1, sRAGE, and hsCRP the independent variables explained only minor percentage of variability of respective dependent variables.

### 3.2. 25(OH)D Status

**Figure 1:**
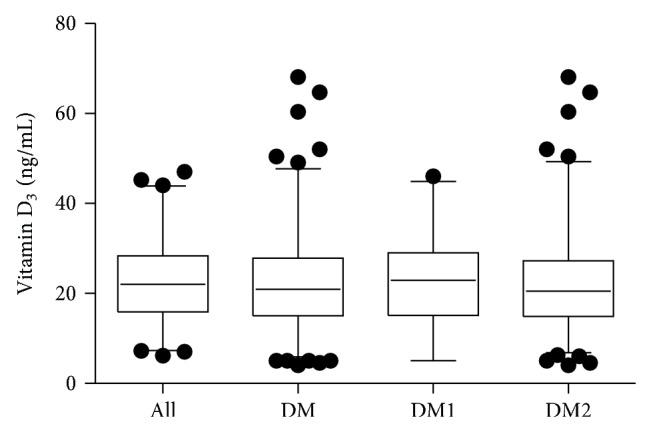
25(OH)D concentration in the controls, diabetic patients (DM), type 1 (DM1) or type 2 (DM2) diabetics. Data are presented as median, interquartile range, and 95% confidence interval (CI). Dots represent outliers beyond 95% CI.

The mean concentrations of 25(OH)D were in the lower range of 25(OH)D insufficiency in the controls and diabetics and did not differ significantly between the groups (Figure 1). Age and presence/absence of diabetes in the whole cohort, or age, duration, and type of diabetes in diabetics showed no significant impact on 25(OH)D levels (Tables 2 and 3).

**Figure 2:**
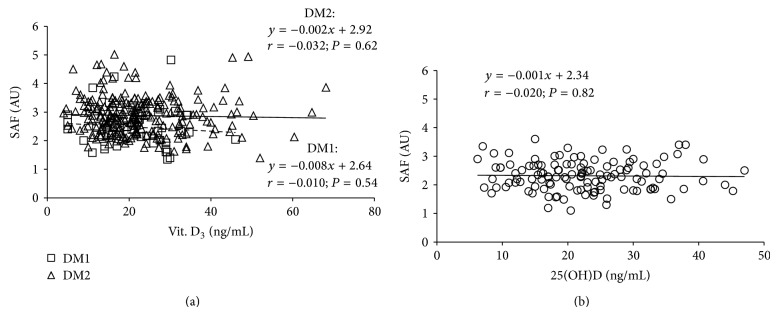
Relationship between skin autofluorescence (SAF) and 25(OH)D levels in type 1 and type 2 diabetics (a) and control subjects (b). DM1: type 1 diabetes mellitus; DM2: type 2 diabetes mellitus; dotted line represents a regression in cohort of type 1 diabetics; solid line represents a regression in cohort of type 2 diabetics.

In the controls no significant relationships between any other tested variable and 25(OH)D levels were revealed (Table 4). In diabetic subjects HbA1c, CML, and sVAP-1 levels correlated inversely, and grip strength directly with 25(OH)D concentrations. Similar to observations in the whole cohort of diabetics, significant associations between HbA1c, CML, sVAP-1, levels, and grip strength on the one hand and 25(OH)D concentration on the other were revealed in type 2 diabetics, while in type 1 diabetics only the relation between CML or grip strength and 25(OH)D was significant (Table 4). 25(OH)D levels showed no significant relationship with SAF either in type 1 and type 2 diabetics (Figure 2(a)) or in the controls (Figure 2(b)).

The prevalence of 25(OH)D deficiency (47% versus 44%), insufficiency (32% versus 35%), and sufficient levels (21% versus 21%) was similar among diabetics and controls (*P*_chi_ = 0.76).

A multivariate analysis using the OPLS-DA model was employed to elucidate which variables contribute to separation between 25(OH)D deficient subjects and those presenting sufficient levels.

**Figure 3:**
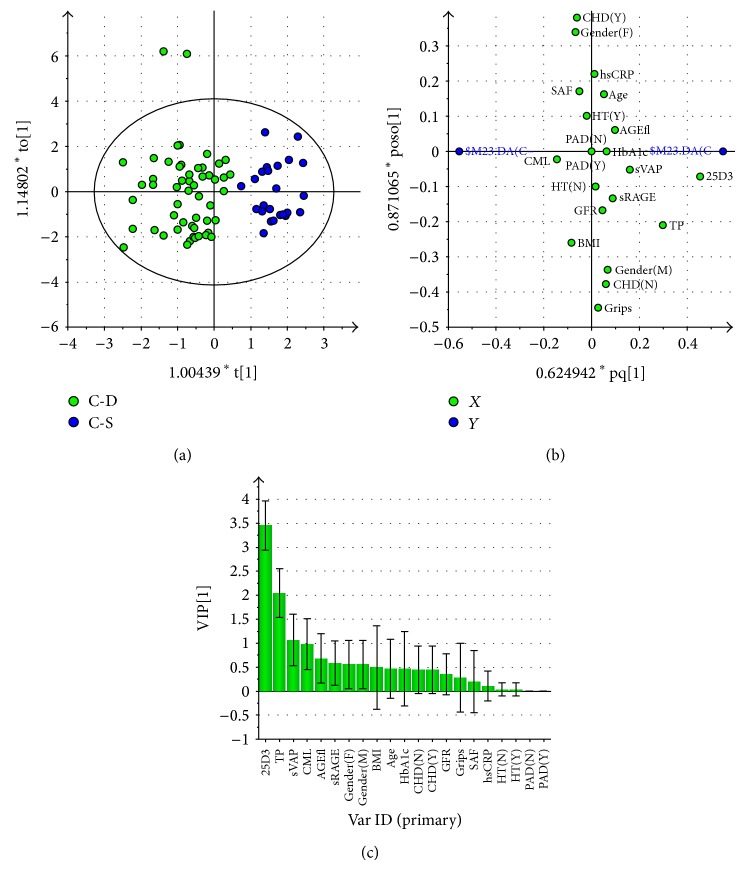
Multivariate analysis data from OPLS-DA model comparing 25(OH)D deficient nondiabetic subjects (25(OH)D <20 ng/mL) with those presenting sufficient levels (25(OH)D >30 ng/mL). (a) Score scatter plot of 25(OH)D deficient controls (C-D, green squares) and those presenting sufficient 25(OH)D levels (C-S, blue squares). Scores are orthogonal (=completely independent from each other), representing new variables summarizing the input of all determined variables (herein gender, presence or absence of comorbidities, age, SAF, and biochemical variables) so that one score vector corresponds to one subject, having its own score vector. Observations situated far outside Hotelling's T2 tolerance ellipse are outliers. Model reveals separation of 25(OH)D deficient and sufficient subjects (separation in direction of *x*-axis). Separation in direction of *y*-axis represents within group variability. (b) Loading scatter plot of 25(OH)D deficient controls and those presenting sufficient 25(OH)D levels. Dummy variables (blue circles) characterize the respective 2 groups categorized according to 25(OH)D levels, deficient group at left, and sufficient one at right side of the plot. Vitamin D_3_ (25D3) adjacent to dummy variable representing 25(OH)D sufficient group represents the most significant component with discriminatory power determining the separation between the groups; being situated in the vicinity of vitamin D sufficient group presenting dummy variable it indicates that it is higher in this group. 25(OH)D deficient subjects also tend to present higher CML levels (positioned in vicinity of respective dummy variable), and lower total protein and sVAP-1 levels (far opposite, right to respective dummy). Variables positioned near to intersect and on *y*-axis are similar in 25(OH)D deficient and sufficient groups and thus do not contribute to between-group separation. (c) Plot of variables of importance contributing to between-group separation among 25(OH)D deficient controls and those presenting sufficient 25(OH)D levels. Plot of variables importance for the projection (VIP) summarizes the importance of the variables both to explain *X* and to correlate with dummy variables (in (a), and (b)). VIP values >1 indicate “important” *X* variables, <0.5 “unimportant” *X* variables, in the “grey interval” (0.5-to-1) the importance depends on the sample size. This plot confirms the OPLS-DA loadings scatter plot (b), showing that the variables adjacent to the origin in the former plot do not contribute to between-group separation significantly. Abbreviations used in (b) and (c): 25D3: 25(OH) vitamin D_3_; TP: plasma total protein concentration; sVAP: soluble vascular receptor adhesion protein-1; CML: N^*ε*^-carboxymethyllysine; AGE-fl: advanced glycation end products associated fluorescence of plasma; sRAGE: soluble receptor for advanced glycation end products; F: female; M: male; BMI: body mass index; HbA1c: glycated hemoglobin A1c; DM1: type 1 diabetes mellitus; DM2: type 2 diabetes mellitus; eGFR: estimated glomerular filtration rate; CHD: coronary heart disease; N: no, absent; Y: yes, present; GFR: estimated glomerular filtration rate; grips: grip strength; SAF: skin autofluorescence; AOPPs: advanced oxidation protein products; hsCRP: high sensitive C-reactive protein; HT: hypertension; PAD: peripheral artery disease.

In the control group a satisfactory separation between 25(OH)D deficient subjects and those with satisfactory levels was obtained (Figure 3(a)). The calculated model described 76% of variability (*R* ^2^) with an acceptable predictivity (*Q* ^2^ = 0.65). Loading scatter plot (Figure 3(b)) and VIP plot (Variables Important for Projection, Figure 3(c)) suggests that 25(OH)D deficient controls present lower total protein (VIP = 2.1) and sVAP-1 (VIP = 1.1) and higher CML (VIP = 1.0) levels. AGE-Fl, sRAGE (lower in 25(OH)D deficient subjects), and BMI (higher in 25(OH)D deficient subjects; VIP values between 0.7 and 0.5) represent variables potentially important for the separation between groups. Moreover, females tended to be more frequent in the 25(OH)D deficient group. Variables listed to the right from BMI on the VIP plot (VIP < 0.5; Figure 3(c)) placed in the vicinity of the intersection of *x*- and *y*-axis and zero *y*-axis on loading scatter plot (Figure 3(b)) do not have discriminatory power in this model.

However, except for 25(OH)D levels, the *t*-test did not indicate significance, only trends corresponding to results of the multivariate analysis (Table 5).

**Figure 4:**
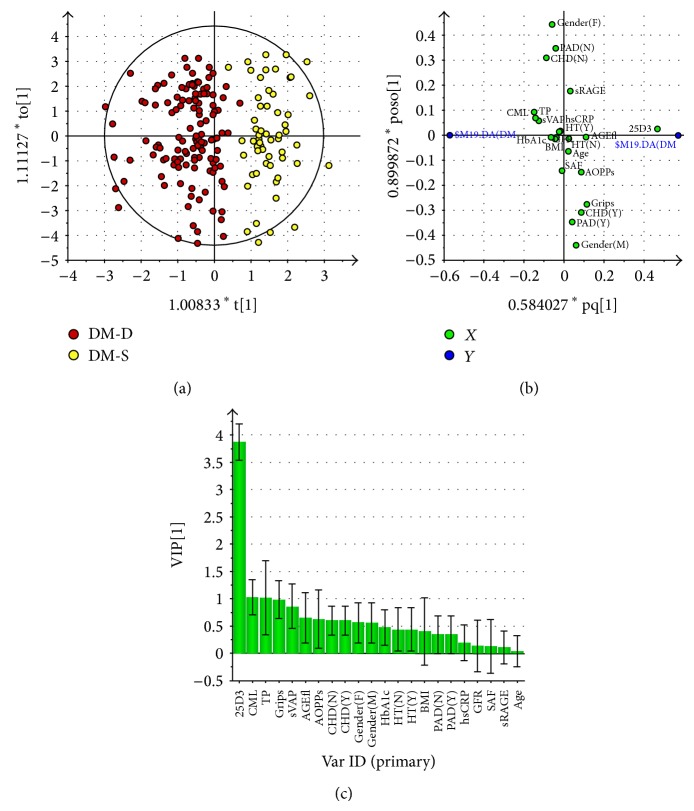
Multivariate analysis data from OPLS-DA model comparing 25(OH)D deficient diabetic patients (25(OH)D <20 ng/mL) with those presenting sufficient levels (25(OH)D >30 ng/mL). (a) Score scatter plot of 25(OH)D deficient diabetic patients (DM-D, red circles) and those presenting sufficient 25(OH)D levels (DM-S, yellow circles). Scores are orthogonal (=completely independent from each other), representing new variables summarizing the input of all determined variables (herein gender, presence or absence of comorbidities, age, SAF, and biochemical variables) so that one score vector corresponds to one subject, having its own score vector. Observations situated far outside Hotelling's T2 tolerance ellipse are outliers. Model reveals separation of 25(OH)D deficient and sufficient diabetic subjects (separation in direction of *x*-axis). Separation in direction of *y*-axis represents within group variability. (b) Loading scatter plot of 25(OH)D deficient diabetic subjects and those presenting sufficient 25(OH)D levels. Dummy variables (blue circles) characterize the respective 2 groups categorized according to 25(OH)D levels, deficient group at left, and sufficient one at right side of the plot. Vitamin D_3_ (25D3) adjacent to dummy variable representing 25(OH)D sufficient group represents the most significant component with discriminatory power determining the separation between the groups; being situated in the vicinity of vitamin D sufficient group presenting dummy variable it indicates that it is higher in this group. 25(OH)D deficient subjects also tend to present higher CML, total protein and sVAP-1 levels (positioned in vicinity of respective dummy variable), and lower AGE-associated fluorescence of plasma, AOPPs and grip strength (far opposite, right to respective dummy). Variables positioned near to intersect and on *y*-axis are similar in 25(OH)D deficient and sufficient groups and thus do not contribute to between-group separation. (c) Plot of variables of importance contributing to between-group separation among 25(OH)D deficient controls and those presenting sufficient 25(OH)D levels. Plot of variables importance for the projection (VIP) summarizes the importance of the variables both to explain *X* and to correlate with dummy variables (in (a), and (b)). VIP values >1 indicate “important” *X* variables, <0.5 “unimportant” *X* variables, in the “grey interval” (0.5-to-1) the importance depends on the sample size. This plot confirms the OPLS-DA loadings scatter plot (b), showing that the variables adjacent to the origin in the former plot do not contribute to between-group separation significantly. Abbreviations used in Figures 3(b) and 3(c): 25D3: 25(OH) vitamin D_3_; TP: plasma total protein concentration; sVAP: soluble vascular receptor adhesion protein-1; CML: N^*ε*^-carboxymethyllysine; AGEfl: advanced glycation end products associated fluorescence of plasma; sRAGE: soluble receptor for advanced glycation end products; F: female; M: male; BMI: body mass index; HbA1c: glycated hemoglobin A1c; DM1: type 1 diabetes mellitus; DM2: type 2 diabetes mellitus; eGFR: estimated glomerular filtration rate; CHD: coronary heart disease; N: no, absent; Y: yes, present; GFR: estimated glomerular filtration rate; grips: grip strength; SAF: skin autofluorescence; AOPPs: advanced oxidation protein products; hsCRP: high sensitive C-reactive protein; HT: hypertension; PAD: peripheral artery disease.

In the diabetic cohort OPLS-DA analysis revealed a satisfactory separation between the 25(OH)D deficient and sufficient subjects (Figure 4(a)), with *R* ^2^ = 0.71 and *Q* ^2^ = 0.64. Loading scatter (Figure 4(b)) and VIP (Figure 4(c)) plots suggest that 25(OH)D deficient diabetics present higher CML, total protein levels, and a weaker grip strength (VIP = 1.0, all). The levels of sVAP-1 (higher, VIP = 0.9) and AGE-Fl (lower, VIP = 0.7) represent variables potentially contributing to separation. 25(OH)D deficient diabetics comprised more females and fewer subjects suffering from CHD i compared to those presenting sufficient 25(OH)D levels. HbA1c, BMI, eGFR, sRAGE, age, and presence of hypertension or PAD did not show discriminatory power (Figures 4(b) and 4(c)).

**Figure 5:**
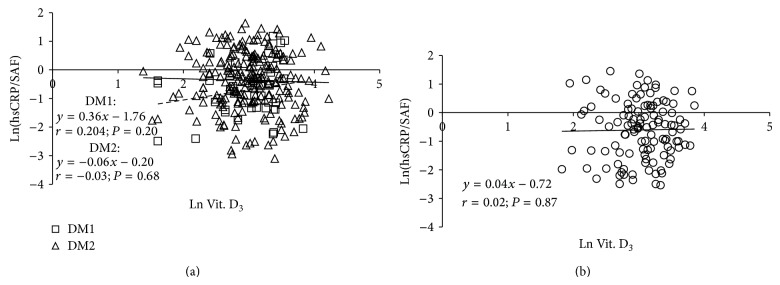
Regression of ln(hsCRP/SAF) over 25(OH)D concentration in type 1 and type 2 diabetics (a) and control subjects (b). DM1: type 1 diabetes mellitus; DM2: type 2 diabetes mellitus; dotted line represents a regression in cohort of type 1 diabetics; solid line represents a regression in cohort of type 2 diabetics.

**Figure 6:**
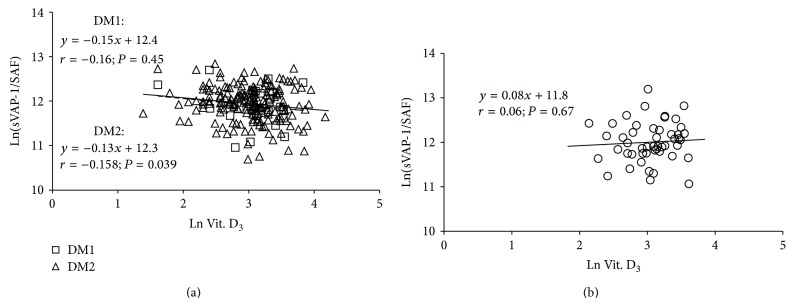
Regression of ln(sVAP-1/SAF) over 25(OH)D concentration in type 1 and type 2 diabetics (a) and control subjects (b). DM1: type 1 diabetes mellitus; DM2: type 2 diabetes mellitus; dotted line represents a regression in cohort of type 1 diabetics; solid line represents a regression in cohort of type 2 diabetics.

Between-group comparison using the *t*-test confirmed the results indicated in multivariate analysis: 25(OH)D deficient diabetics presented higher CML, sVAP-1, and total proteins levels; lower AGE-Fl and grip strength and lower prevalence of CHD (Table 5). The impact of inflammation and SAF to the 25(OH)D levels was further approximated as relationship between Ln(hsCRP/SAF) and 25(OH)D. No significant association was revealed either in diabetics or in the controls (Figures 5(a) and 5(b)). However, Ln(sVAP-1/SAF) correlated inversely in all diabetic patients (*y* = −0.006*x* − 0.006; *r* = 0.148; *P* = 0.040) on account of the type 2 diabetics (Figure 4(a)). In type 1 diabetics and control subjects significance was not reached (Figures 6(a) and 6(b)).

### 3.3. Markers of Advanced Glycation End Products

**Figure 7:**
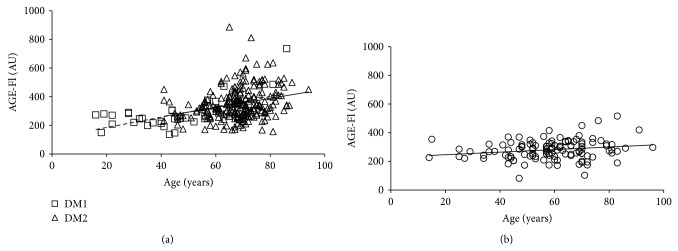
Age-dependent rise in AGE-specific fluorescence of plasma (AGE-Fl) in type 1 and type 2 diabetics (a) and controls (b). DM1: type 1 diabetes mellitus; DM2: type 2 diabetes mellitus; dotted line represents a regression in cohort of type 1 diabetics; solid line represents a regression in cohort of type 2 diabetics; AU: arbitrary units.

**Figure 8:**
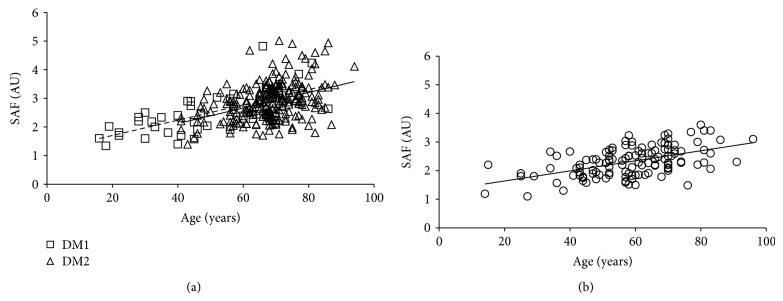
Age-dependent rise in skin autofluorescence (SAF) in controls (a) and type 1 and type 2 diabetics (b). DM1: type 1 diabetes mellitus; DM2: type 2 diabetes mellitus; dotted line represents a regression in cohort of type 1 diabetics; solid line represents a regression in cohort of type 2 diabetics; AU: arbitrary units.

In type 1 and 2 diabetics an age-dependent rise in AGE-associated fluorescence of plasma was described by almost parallel lines (*y* = 3.6 × age + 113, *r* = 0.636, *P* < 0.001 and *y* = 3.2 × age + 132, *r* = 0.272, *P* < 0.001), (Figure 7(a)) and that of SAF by fully parallel lines (*y* = 0.027 × age + 1.17, *r* = 0.671, *P* < 0.001 and *y* = 0.027 × age + 1.03, *r* = 0.397, *P* < 0.001, resp.) (Figure 8(a)). GLM revealed significant impact of age and diabetes duration but not of type of diabetes on SAF and AGE-Fl (Table 3).

In type 1 diabetics multivariate analysis (with age, BMI, eGFR, HbA1c, hsCRP, SAF, CML, sRAGE, sVAP-1, and duration of diabetes entered as independent variables) selected only sVAP-1 (corrected model *P* = 0.005, *R* ^2^ : 78%; *P*_sVAP-1_ = 0.028, *β* = −0.51, SE = 0.19) as a significant independent contributor to AGE-Fl. In type 2 diabetic subjects eGFR was selected as an independent significant contributor to AGE-Fl (corrected model *P* < 0.001, *R* ^2^: 38%; *P*_eGFR_ < 0.001, *β* = −0.006, SE = 0.001).

In a similar setting for SAF levels GLM selected age (*P*_age_ = 0.019, *β* = 0.90, SE = 0.31), sVAP-1 (*P* = 0.047, *β* = 0.99, SE = 0.41), and sRAGE levels (*P* = 0.001, *β* = −1.13, SE = 0.20; corrected model *P* = 0.006, *R* ^2^: 84%) as significant associated factors of type 1 diabetics. In type 2 diabetic subjects age (*P* = 0.006, *β* = 1.01, SE = 0.36), eGFR (*P* = 0.022, *β* = −0.005, SE = 0.002), sRAGE levels (*P* = 0.016, *β* = −0.23, SE = 0.09), sVAP-1 (*P* = 0.014, *β* = 0.27, SE = 0.11), and CML (*P* = 0.050, *β* = −0.21, SE = 0.11) appeared to be associated independently with SAF levels (corrected model *P* < 0.001, *R* ^2^: 37%).

In comparison with the diabetics, the age-dependent rise in SAF (*y* = 0.018 × age + 1.28, *r* = 0.520, *P* < 0.001) and AGE-associated fluorescence of plasma (*y* = 0.9 × age + 228, *r* = 0.202, *P* = 0.028) was much slower in the controls (Figures 7(b) and 8(b)).

GLM did not select any independent variable association independently and significantly either to AGE-Fl or to SAF in the controls.

The correction for 25(OH)D did not change the results nor was 25(OH)D selected as significant contributor if forced into either model.

## 4. Discussion

This is one of the few studies examining in diabetic subjects the relationship between vitamin D status and AGE accumulation in plasma and skin as well as the AGE-associated biomarkers of microinflammation and oxidative stress. Surprisingly we found no association between vitamin D status and SAF or plasma AGE-Fl. Among the markers of microinflammation/oxidative stress an inverse link between vitamin D and sVAP-1 in the type 2 diabetics could be shown.

### 4.1. Vitamin D Status

In our study the controls presented mean vitamin D levels within the lower range of vitamin D insufficiency and averaging 22.3 ng/mL. This finding corresponds to concentrations reported for the Central European general population (17-to-33 ng/mL in mean) from cities in similar latitude as Würzburg (reviewed in [51]). They are higher than those reported for a large cohort of orthopaedic patients in Germany (18.8 ng/mL in mean in summer and 16.1 ng/mL in winter) [52].

In our diabetic subjects the prevalence of hypovitaminosis D was of the same degree as in the controls. In other studies the prevalence of low vitamin D levels was higher in diabetics and individuals with prediabetes compared to nondiabetic controls [53–56]. Consequently a low vitamin D status was assumed to be involved in the development and also progression of type 2 diabetes. This hypothesis was supported by various experimental and clinical studies which showed an enhanced insulin resistance and/or an impaired secretion of insulin in the pancreatic beta cells in the presence of hypovitaminosis D [57, 58]. In line with the Tromso study [59] and Zoppini et al. [60], we found in our diabetics an inverse relationship between vitamin D and the HbA1c concentrations. However, this is not a causal relationship since in a meta-analysis of numerous studies vitamin D supplementation was without effect on the disturbed glucose homeostasis [61]. Furthermore in a recent genetic study no proof for a relationship between vitamin D deficiency and diabetes was found [62].

### 4.2. Vitamin D Status and BMI

We did not reveal a significant association between vitamin D levels and BMI, in either diabetics or nondiabetic controls; however, multivariate analysis suggested that a higher BMI might be inversely associated with vitamin D status in nondiabetics. Cholecalciferol is the dominant metabolite and distributed in adipose tissue [63]. Its accumulation in fat cells probably results from its trapping. There is a significant positive association between 25(OH)D concentration in subcutaneous white adipose tissue and serum [64]. A meta-analysis of 21 studies reported that each unit of increase in BMI (kg/m^2^) associates with 1.15% lower plasma concentrations of 25(OH)D [65]. Further data suggest that reduction of weight and consequently of fat in overweight and obese subjects is not associated with significant changes in white adipose tissue or circulating vitamin D_3_ levels [64].

### 4.3. Advanced Glycation End Products

In our controls the age-dependent rise of SAF levels corresponded well with those reported for the general Dutch population [66]. As expected, in our diabetics, the age-dependent rise of SAF was much steeper than in the nondiabetic controls. These data correspond well with studies from Netherlands [67, 68] and from the Czech Republic [69]. In contrast, a large Australian study reported only a tendency towards higher SAF values [15]. The reasons are not clear, but differences in the characteristics of the controls (such as age or presence of comorbidities) and of the diabetics (duration, treatment modalities, and presence of complications and comorbidities) might be important.

Interestingly in our type 1 and type 2 diabetics the age-dependent rise of SAF was of the same magnitude and the SAF levels were directly related to duration of the disease (similar between both diabetic groups). No relationships were found to haemoglobin A1c. Also plasma Fl-AGE showed an age-dependent rise in our controls and the diabetics which is in accordance with data of Kalousová et al. [70].

### 4.4. Missing Interactions of Vitamin D and Advanced Glycation End Products

Since in vitro and animal studies showed that vitamin D application lowers the toxic effects of AGEs and decreases their formation [48, 49], we were particularly interested in potential relationships between both factors. We expected that low levels of vitamin D could be associated with an enhanced AGE formation, while, in the presence of sufficient vitamin D, lower concentrations of AGEs should occur. Surprisingly, we did not found any link between vitamin D and SAF.

Also plasma Fl-AGE and vitamin D levels were not interrelated. However, multivariate analysis using the OPLS-DA model suggested that vitamin D_3_ deficient subjects tend to present lower AGE-associated fluorescence of plasma, regardless of presence or absence of diabetes. In a study in elderly type 2 diabetics no relationship between vitamin D_3_ levels and plasma concentration of Fl-AGEs was found [55].

The missing link between SAF and vitamin D also rules out the possibility that the cutaneous AGE accumulation hinders the photoconversion of the provitamin D into vitamin D. In line with this assumption, repeated UVB radiation in hemodialysis patients was associated with a marked increase of vitamin D_3_ status despite the high levels of skin AGEs in these patients [71].

### 4.5. Markers of Inflammation and Oxidative Stress

In the whole cohort of the diabetics the plasma levels of hs-CRP and sVAP-1 were significantly elevated. Unexpectedly, this rise was on the account of the type 2 diabetics, while in type 1 diabetics these markers of the inflammatory pathway were not enhanced. This observation is surprising with regard to the identical elevation of the HbA1c levels in both groups. This disparate pathobiochemistry may be explained by the different aetiology and pathophysiology of both diabetic states as also proposed by Kalousová et al. [70]. Type 1 diabetes mellitus is an immunological disease and characterized by dysfunction of the pancreatic *β*-cells. It usually develops in younger age in the absence of obesity, insulin resistance, and hypertension. In contrast, type 2 diabetes mellitus represents an insulin-resistant state and manifests in the majority of patients with a metabolic syndrome in the middle or higher age. Central obesity favors the development of dyslipidemia, microinflammation, and oxidative stress [72, 73] as well as hypertension [74]. Correspondingly, our type 2 diabetics were older, presented 1.4-fold higher incidence of hypertension and a higher BMI as compared to type 1 diabetics.

The sVAP-1 levels were elevated in hypovitaminosis D and showed an independent inverse association with the 25(OH)D concentrations in both diabetic cohorts. The augmented sVAP-1 levels might reflect involvement of oxidative stress and/or microinflammation in part due to AGE accumulation. sVAP-1, known also as semicarbazide-sensitive amine oxidase (SSAO, EC 1.4.3.21), represents a molecule with a dual action: it favors lymphocyte adhesion to damaged endothelium and plays a role in the monoamine detoxification metabolizing primary amines into corresponding aldehydes, generating H_2_O_2_ and ammonia [75]. Increased SSAO activity in diabetes may result from enhanced SSAO substrates such as methylamine or aminoacetone.

It remains unclear whether the association of low vitamin D_3_ and elevated sVAP-1 levels is a coincidence or causally related to proinflammatory consequences of hypovitaminosis D. In the latter case an augmented sVAP-1 could result from release from endothelial cells (following the shedding by metalloproteinases) or induction of the VAP-1/SSAO gene expression. In diabetes the conversion of aminoacetone by SSAO to methylglyoxal is increased and has been claimed to be related to induction of insulin resistance and development of diabetic complications [76–78]. In humans the hyperglycemia-induced rise of circulating sVAP-1 levels directly correlates with the plasma AGE concentration [79]. Moreover, sVAP-1 is associated with subclinical atherosclerotic manifestations and an increased risk of cardiovascular events and mortality rate [80].

Since both AGE accumulation [81] and vitamin D deficiency [82] may be associated with impaired muscle strength we performed the hand-grip test in the controls and diabetics. In our study we did not observe an association between elevated AGEs and impaired grip strength in control group or in diabetics. However, in contrast to the controls, in diabetic patients (type 1 and type 2) with hypovitaminosis D (insufficient and deficient vitamin D levels), we noticed impaired grip strength.

In our study, potential confounding factors to the toxic effects of AGEs and hypovitaminosis D have to be considered. First of all the impact of tight blood glucose control has to be mentioned. The mean HbA1c level of 7.1 demonstrates that in most patients glycemia was well controlled. All hypertensive patients including the nondiabetic controls were treated with either ACE-inhibitors or angiotensin II receptor blockers. These compounds exert anti-inflammatory, antioxidative, and anti-AGE forming effects as demonstrated in in vitro studies, animal models, and clinical trials [83–85]. Furthermore the frequently prescribed statins were shown to reduce microinflammation and oxidative stress [86–88] as well as the formation of AGEs [89], in part by increasing soluble RAGE [90]. Interestingly certain statins may enhance the formation of vitamin D_3_ [91] which could explain the missing difference of vitamin D levels in our nondiabetic and diabetic cohorts. A very potent drug with anti-inflammatory actions is metformin which was administered to most of the type 2 diabetic patients [92, 93] except in patients with creatinine clearance less than 60 mL/min.

Taken together, in our diabetic patients, the vitamin D_3_ deficiency was not unequivocally and expressively associated with markers of microinflammation and oxidative stress. This could suggest that the postulated anti-inflammatory action of vitamin D is rather limited or may even be absent under certain conditions. In fact in various inflammatory states the vitamin deficiency was not the cause, but a consequence of the disease (elective knee arthroplasty [94], critically ill patients [95]). Correspondingly in various controlled studies (in contrast to observational investigations), supplementation with vitamin D did not improve the microinflammation and oxidative stress [62]. However, in patients with chronic kidney disease, hypovitaminosis D is frequently associated with secondary hyperparathyroidism which is suppressed by vitamin D supplementation [96]. Although still needing placebo-controlled studies, vitamin D supplementations in elderly people “seemed to decrease mortality” [97].

Summarizing, our cross-sectional study suggests that in diabetic subjects hypovitaminosis D is not associated with enhanced AGE accumulation and sufficient vitamin D levels are not linked with a lower AGE accumulation. Moreover, we conclude that an excessive rise of skin AGEs does not interfere with dermal vitamin D_3_ formation. The relationships between vitamin D_3_ deficiency and markers of inflammation showed a different pattern. We found higher levels of s-VAP 1 in hypovitaminosis D, but hs-CRP levels were unchanged. These data suggest that hypovitaminosis D seems to be of limited importance for development of microinflammation and accumulation of AGEs. With regard to equivocal results of our cross-sectional study, controlled longitudinal studies focusing on the effects of vitamin D supplementation on skin and plasma AGE and markers of microinflammation/oxidative stress are needed to elucidate a potential relationship between vitamin D status and AGE accumulation and their interaction in potentiating of toxic effects.

## Figures and Tables

**Table 1:** Cohort characteristics.

	Controls	All DM patients	*P*	DM1 patients	DM2 patients	*P*
*N* (%)	121	276		43 (16%)	233 (84%)	
Males/females (*n*; %)	60/61 (50%/50%)	160/116 (58%/42%)	0.12^chi^	23/20 (53%/47%)	137/96 (59%/41%)	0.52^chi^
Age (years)	*58.6 ± 15.1 *	*65.0 ± 13.4 *	***<0.001***	*48.6 ± 1.8 *	*68.0 ± 9.6 *	***<0.001***
DM duration (yrs)	—	*15.1 ± 10.7 *	—	*19.5 ± 13.2 *	*15.0 ± 10.1 *	*0.10 *
BMI (kg/m^2^)	27.2 ± 3.3	30.5 ± 6.1	**<0.001**	25.4 ± 3.7	31.4 ± 6.0	**<0.001**
Total protein (g/L)	72 ± 7	72 ± 7	0.71	71 ± 5	72 ± 7	0.24
eGFR (mL/min/1.73 m^2^)	84 ± 16	71 ± 25	**<0.001**	83 ± 26	69 ± 24	**<0.001**
HbA1c (%)	*5.5 ± 0.3 *	*7.1 ± 1.1 *	**<0.001**	*7.1 ± 1.1 *	*7.1 ± 1.1 *	0.98
Fl-AGEs (AU)	*281 ± 67 *	*341 ± 112 *	***<0.001***	*289 ± 104 *	*350 ± 111 *	***<0.001***
SAF (AU)	2.3 ± 0.5	2.8 ± 0.7	**<0.001**	2.5 ± 0.7	2.9 ± 0.7	**<0.001**
CML (ng/mL)	*1045 ± 368 *	*1023 ± 393 *	*0.71 *	*1125 ± 940 *	*1008 ± 385 *	*0.12 *
sRAGE (pg/mL)	*989 ± 376 *	*936 ± 497 *	*0.23 *	*1133 ± 638 *	*922 ± 474 *	*0.30 *
sVAP-1 (ng/mL)	409 ± 166	462 ± 172	***0.049***	415 ± 138	469 ± 176	0.16
hsCRP (mg/L)	*2.0 ± 2.0 *	*2.7 ± 2.4 *	***0.001***	*1.9 ± 1.9 *	*2.9 ± 2.4 *	***0.004***
Grip strength (pounds)	*84 ± 31 *	*75 ± 25 *	***0.026***	*85 ± 32 *	*74 ± 23 *	*0.09 *
^*^Hypertension (N/Y; %)	88/33 (73%/27%)	96/167 (37%/63%)	**<0.001** ^chi^	23/13 (53%/47%)	73/154 (32%/68%)	**<0.001** ^chi^
^*^PAD (N/Y; %)	119/2 (98%/2%)	220/44 (83%/17%)	**<0.001** ^chi^	34/2 (94%/6%)	186/42 (82%/18%)	0.054^chi^
^*^CHD (N/Y; %)	117/4 (97%/3%)	213/51 (81%/19%)	**<0.001** ^chi^	33/3 (92%/8%)	180/48 (79%/21%)	0.07^chi^
^*^Total comorbidities (N/Y; %)	87/34 (72%/28%)	74/190 (28%/72%)	**<0.001** ^chi^	22/114 (61%/39%)	52/176 (23%/77%)	**<0.001** ^chi^

DM1: type 1 diabetes mellitus; DM2: type 2 diabetes mellitus; BMI: body mass index; eGFR: estimated glomerular filtration rate; HbA1c: haemoglobin A1c; AGE-Fl: advanced glycation end products associated fluorescence of plasma; CML: N^*ε*^-carboxymethyllysine; SAF: skin autofluorescence; sVAP-1: soluble vascular receptor adhesion protein-1; sRAGE: soluble receptor for advanced glycation end products; hsCRP: high sensitive C-reactive protein; Y: yes; N: no; PAD: peripheral artery disease; CHD: coronary heart disease; chi: chi-square; ^*^data missing from 12 subjects.

**Table 2:** Multiple regression, effect of ageing, and presence/absence of DM on selected independent variables.

	*25(OH)D *	*HbA*1*c *	*AGE-Fl *	*CML *	SAF	sVAP-1	*sRAGE *	*hsCRP *	*Grips *
Corr. m.	0.84	**0.001**	**0.001**	0.93	**0.001**	**0.045**	**0.019**	**0.001**	**0.001**
Intercept	0.001	**0.001**	**0.001**	0.001	**0.001**	**0.001**	**0.002**	**0.011**	**0.001**
*Age *	0.83	0.06	**0.001**	0.90	**0.001**	0.23	**0.006**	**0.002**	**0.001**
DM st.	0.62	**0.001**	**0.001**	0.69	**0.001**	0.10	0.20	**0.007**	0.94
*R* ^2^	−0.01	0.35	0.15	−0.01	0.31	0.01	0.03	0.05	0.17

25(OH)D: 25(OH)D_3_; HbA1c: haemoglobin A1c; AGE-Fl: advanced glycation end products associated fluorescence of plasma; CML: N^*ε*^-carboxymethyllysine; SAF: skin autofluorescence; sVAP-1: soluble vascular receptor adhesion protein-1; sRAGE: soluble receptor for advanced glycation end products; hsCRP: high sensitive C-reactive protein; corr. M.: corrected model; DM st.: diabetic status, classified 0/1 as absence/presence; *italics:* due to not normal distribution statistics performed on logarithmically transformed data. In case of sVAP-1, sRAGE, and hsCRP model was significant although age and presence of diabetes explained <5% in their variability (*R* ^2^).

**Table 3:** Multiple regression, effect of ageing, and duration of diabetes and DM type on selected independent variables.

	*25(OH)D *	*eGFR *	*AGE-Fl *	*CML *	SAF	sVAP-1	*sRAGE *	*hsCRP *	*Grips *
Corr. M.	0.75	**0.001**	**0.001**	**0.049**	**0.001**	**0.002**	**0.004**	**0.016**	**0.001**
Intercept	0.001	**0.001**	**0.001**	**0.001**	**0.001**	**0.001**	**0.001**	0.44	**0.001**
*DM dur*.	0.92	**0.001**	**0.001**	0.42	**0.003**	**0.027**	**0.008**	0.89	**0.001**
*Age *	0.28	**0.001**	**0.001**	0.09	**0.001**	0.09	0.29	0.28	0.08
DM type	0.52	0.84	0.24	0.60	0.71	0.28	0.38	0.07	0.47
*R* ^2^	−0.01	0.27	0.20	0.03	0.24	0.06	0.05	0.03	0.17

25(OH)D: 25(OH)D_3_; eGFR: estimated glomerular filtration rate; AGE-Fl: advanced glycation end products associated fluorescence of plasma; CML: N^*ε*^-carboxymethyllysine; SAF: skin autofluorescence; sVAP-1: soluble vascular receptor adhesion protein-1; sRAGE: soluble receptor for advanced glycation end products; hsCRP: high sensitive C-reactive protein; corr. M.: corrected model; DM type: type 1 or type 2 diabetes; *italics*: due to not normal distribution statistics performed on logarithmically transformed data. In case of CML, sRAGE and hsCRP model was significant although age and type of diabetes explained ≤5% in their variability (*R* ^2^).

**Table 4:** Pearson correlation coefficients independent variables to 25(OH)D_3_.

	Controls		DM patients		DM1		DM2	
	*r*	*P*	*r*	*P*	*r*	*P*	*r*	*P*
ln⁡age	0.107	0.24	−0.065	0.28	−0.097	0.54	−0.077	0.24
ln⁡DM duration	NA	NA	NA	NA	0.201	0.20	−0.057	0.39
BMI	0.049	0.65	−0.073	0.23	0.085	0.59	−0.105	0.11
eGFR	0.082	0.50	0.051	0.42	0.028	0.87	0.066	0.34
ln⁡HbA1c	0.060	0.62	−0.122	**0.043**	−0.025	0.087	−0.135	**0.039**
ln⁡Fl-AGEs	0.072	0.44	0.099	0.10	0.087	0.58	0.104	0.12
ln⁡CML	−0.185	0.20	−0.197	**0.006**	−0.444	**0.026**	−0.177	**0.020**
ln⁡sRAGE	−0.062	0.67	−0.027	0.71	−0.319	0.12	−0.007	0.92
sVAP-1	0.040	0.79	−0.199	**0.005**	0.263	0.20	−0.184	**0.016**
ln⁡hsCRP	0.004	0.97	−0.019	0.76	0.150	0.35	−0.059	0.39
ln⁡Grip	0.112	0.30	0.196	**0.002**	0.506	**0.003**	0.141	**0.037**

DM: diabetes mellitus; DM1: type 1 diabetes mellitus; DM2: type 2 diabetes mellitus; ln: logarithmically transformed data; BMI: body mass index; eGFR: estimated glomerular filtration rate; HbA1c: haemoglobin A1c; AGE-Fl: advanced glycation end products associated fluorescence of plasma; CML: N^*ε*^-carboxymethyllysine; sRAGE: soluble receptor for advanced glycation end products; sVAP-1: soluble vascular receptor adhesion protein-1; hsCRP: high sensitive C-reactive protein.

**Table 5:** Pertinent data of the controls and diabetic patients with 25(OH)D_3_ deficiency (25(OH)D_3_ <20 ng/mL) and sufficient levels (25(OH)D_3_ >30 ng/mL).

	Controls			DM subjects		
	25(OH)D <20 ng/mL	25(OH)D >30 ng/mL	*P*	25(OH)D <20 ng/mL	25(OH)D >30 ng/mL	*P*
*N*	53	25	NA	130	58	NA
M/F (*n*; %)	23/30 (43%/57%)	15/10 (60%/40%)	0.17^chi^	71/59 (55%/45%)	39/19 (67%/33%)	0.10^chi^
Age (years)	*58.5 ± 17.1 *	*62.6 ± 13.5 *	*0.23 *	*65.9 ± 13.3 *	*65.5 ± 11.1 *	0.94
DM duration (yrs)	NA	NA		*15.5 ± 10.7 *	*14.7 ± 10.6 *	0.63
BMI (kg/m^2^)	*27.6 ± 3.6 *	*28.6 ± 3.5 *	*0.33 *	*31.2 ± 7.1 *	*29.7 ± 4.6 *	*0.18 *
Total protein (g/L)	74 ± 8	70 ± 6	0.17	74 ± 7	70 ± 7	**0.015**
eGFR (mL/min/1.73 m^2^)	83 ± 12	84 ± 12	*0.71 *	70 ± 26	69 ± 23	*0.70 *
25(OH)D_3_ (ng/mL)	*14.4 ± 3.9 *	*35.5 ± 4.8 *	***<0.001***	*13.9 ± 3.9 *	*38.4 ± 8.7 *	***<0.001***
HbA1c (%)	*5.5 ± 0.3 *	*5.5 ± 0.3 *	*0.53 *	*7.2 ± 1.2 *	*6.9 ± 1.0 *	*0.17 *
Fl-AGEs (AU)	*272 ± 181 *	*291 ± 344 *	*0.06 *	*329 ± 108 *	*368 ± 139 *	***0.05***
SAF (AU)	2.3 ± 0.5	2.3 ± 0.5	0.85	2.9 ± 0.7	2.8 ± 0.8	0.16
CML (ng/mL)	*1098 ± 398 *	*930 ± 391 *	*0.27 *	*1090 ± 433 *	*899 ± 236 *	*0.130 *
sRAGE (pg/mL)	*988 ± 400 *	*1070 ± 404 *	*0.53 *	*947 ± 550 *	*949 ± 493 *	*0.89 *
hsCRP (mg/L)	*2.1 ± 2.4 *	*1.6 ± 1.4 *	*0.85 *	*2.8 ± 2.6 *	*2.5 ± 2.0 *	*0.59 *
sVAP-1 (ng/mL)	*406 ± 152 *	*419 ± 177 *	*0.38 *	*488 ± 174 *	*430 ± 181 *	*0.040 *
Grip strength (pounds)	*81 ± 33 *	*84 ± 26 *	*0.50 *	*70 ± 22 *	*80 ± 23 *	**0.007**
^*^Hypertension (N/Y; %)	38/15 (72%/28%)	17/8 (68%/32%)	0.74^chi^	39/84 (32%/68%)	23/33 (41%/59%)	0.22^chi^
^*^PAD (N/Y; %)	53/0 (100%/0%)	24/1 (96%/4%)	0.70^chi^	108/15 (88%/12%)	46/10 (82%/18%)	0.31^chi^
^*^CHD (N/Y; %)	51/2 (96%/4%)	24/1 (96%/4%)	0.56^chi^	102/21 (83%/17%)	40/16 (71%/29%)	0.08^chi^
^*^Total comorb. (N/Y; %)	38/15 (72%/28%)	17/8 (68%/32%)	0.74^chi^	34/89 (28%/72%)	18/40 (29%/71%)	0.64^chi^

DM: diabetes mellitus; M: males; F: females; BMI: body mass index; eGFR: estimated glomerular filtration rate; HbA1c: haemoglobin A1c; AGE-Fl: advanced glycation end products associated fluorescence of plasma; AU: arbitrary units; SAF: skin autofluorescence; CML: N^*ε*^-carboxymethyllysine; sRAGE: soluble receptor for advanced glycation end products; hsCRP: high sensitive C-reactive protein; sVAP-1: soluble vascular receptor adhesion protein-1; Y: yes; N: no; PAD: peripheral artery disease; CHD: coronary heart disease; comorb.: comorbidities; chi: chi-square; ^*^data from 7 diabetics not available.

## Abbreviations

25(OH)D: 25-Hydroxyvitamin D (calcidiol)
AGEs: Advanced glycation end products
AGE-Fl: Advanced glycation end products associated fluorescence of plasma
ANOVA: Analysis of variance
CHD: Coronary heart disease
BMI: Body mass index
CKD: Chronic kidney disease
CML: N^*ɛ*^-Carboxymethyllysine
GLM: General Linear Model
HbA1c: Haemoglobin A1c
eGFR: Estimated glomerular filtration rate
hs-CRP: High sensitive C-reactive protein
HT: Hypertension
Ln: Logarithm
NF-*κ*B: Nuclear factor kappa-B
NO: Nitric oxide
OPLS-DA: Orthogonal projections to latent structures discriminant analysis
PAD: Peripheral artery disease
RAGE: Receptor for advanced glycation end products
sRAGE: Soluble receptor for advanced glycation end products
SAF: Skin advanced glycation end products associated autofluorescence
sVAP-1: Soluble vascular adhesion protein-1
TGF-*β*-1: Transforming growth factor-*β*-1
VIP: Variables importance for the projection.
